# Supplementary material for: Th2-dependent STAT6-regulated genes in intestinal epithelial cells mediate larval trapping during secondary Heligmosomoides polygyrus bakeri infection
Source: PLoS Pathog. 2023 Apr 5;19(4):e1011296. doi: 10.1371/journal.ppat.1011296 (PMC10109486; doi:10.1371/journal.ppat.1011296)
Supplement: S1 Table — (PDF) [file ppat.1011296.s001.pdf]

S1 Table. Primer sequences used for qRT-PCR analyses.

Gene	5‘- 3‘
Arg1-fw	CTCGCAAGCCAATGTACACG
Arg1-rv	GTATGACGTGAGAGACCACG
Arg1del-fw	GGATTGGCAAGGTGATGG
Arg1del-rv	TCAGTCCCTGGCTTATGG
Chil3-fw	CACAGGTCTGGCAATTCTTC
Chil3-rv	GCTTCATAGTCACGCAAGTC
Hprt-fw	GTT GGA TAC AGG CCA GAC TTT GTT
Hprt-rv	GAG GGT AGG CTG GCC TAT AGG CT
Lh2-fw	CGCCAGTCAGGAAGATCTGGTC
Lh2-rv	CTCCAGAATTCAGGTAGCGTTTCCC
Mmp12-fw	TTGCATTTGGAGCTCACGGAGACT
Mmp12-rv	TCAAGGATGGGGGTTTCACTGGG
Mrc1-fw	GCAAACATTGGGCAGAAGG
Mrc1-rv	AGGAAACGGGAGAACCATC
Pdcd1lg2-fw	AAGCCTCAGCCTAGCAGAAAC
Pdcd1lg2-rv	CAGATCCTTCCAACTCCCAAGAC
Pla2g1b-fw	CTCGGGCCGTGTGGCAGTTC
Pla2g1b-rv	TGCCGAGCCAGAGCACGAGT
Retnla-fw	CTGCTGGGATGACTGCTAC
Retnla-rv	CTGGGTTCTCCACCTCTTC
Retnlb-fw	GGAGAGTGAATCTGCTCTTAGGGGA
Retnlb-rv	AGCCATAGCCACAAGCACATCC
