# Supplementary material for: Th2-dependent STAT6-regulated genes in intestinal epithelial cells mediate larval trapping during secondary Heligmosomoides polygyrus bakeri infection
Source: PLoS Pathog. 2023 Apr 5;19(4):e1011296. doi: 10.1371/journal.ppat.1011296 (PMC10109486; doi:10.1371/journal.ppat.1011296)
Supplement: S1 Fig — (PDF) [file ppat.1011296.s002.pdf]

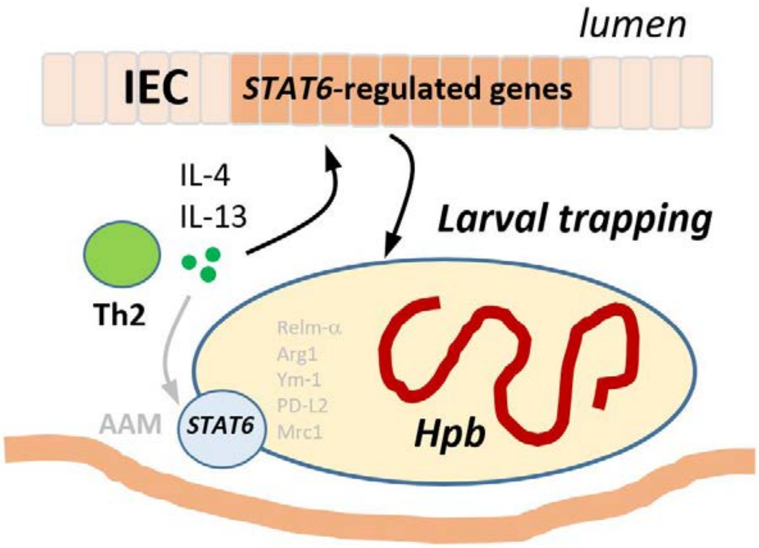

S1 Fig. Graphical abstract. During secondary Heligmosomoides polygyrus bakeri (Hpb) infection Th2 cell-derived IL-4/IL-13 is required to activate STAT6-regulated genes in intestinal epithelial cells (IEC) which then mediate larval trapping in granulomas in the submucosa of the small intestine. Expression of Arginase 1 (Arg1) is dispensable and other STAT6-regulated genes in alternatively activated macrophages (AAM) are not sufficient for larval trapping.
