# Supplementary material for: Th2-dependent STAT6-regulated genes in intestinal epithelial cells mediate larval trapping during secondary Heligmosomoides polygyrus bakeri infection
Source: PLoS Pathog. 2023 Apr 5;19(4):e1011296. doi: 10.1371/journal.ppat.1011296 (PMC10109486; doi:10.1371/journal.ppat.1011296)
Supplement: S2 Fig — AAMs in IL-4c-treated Mac-STAT6 mice. (PDF) [file ppat.1011296.s003.pdf]

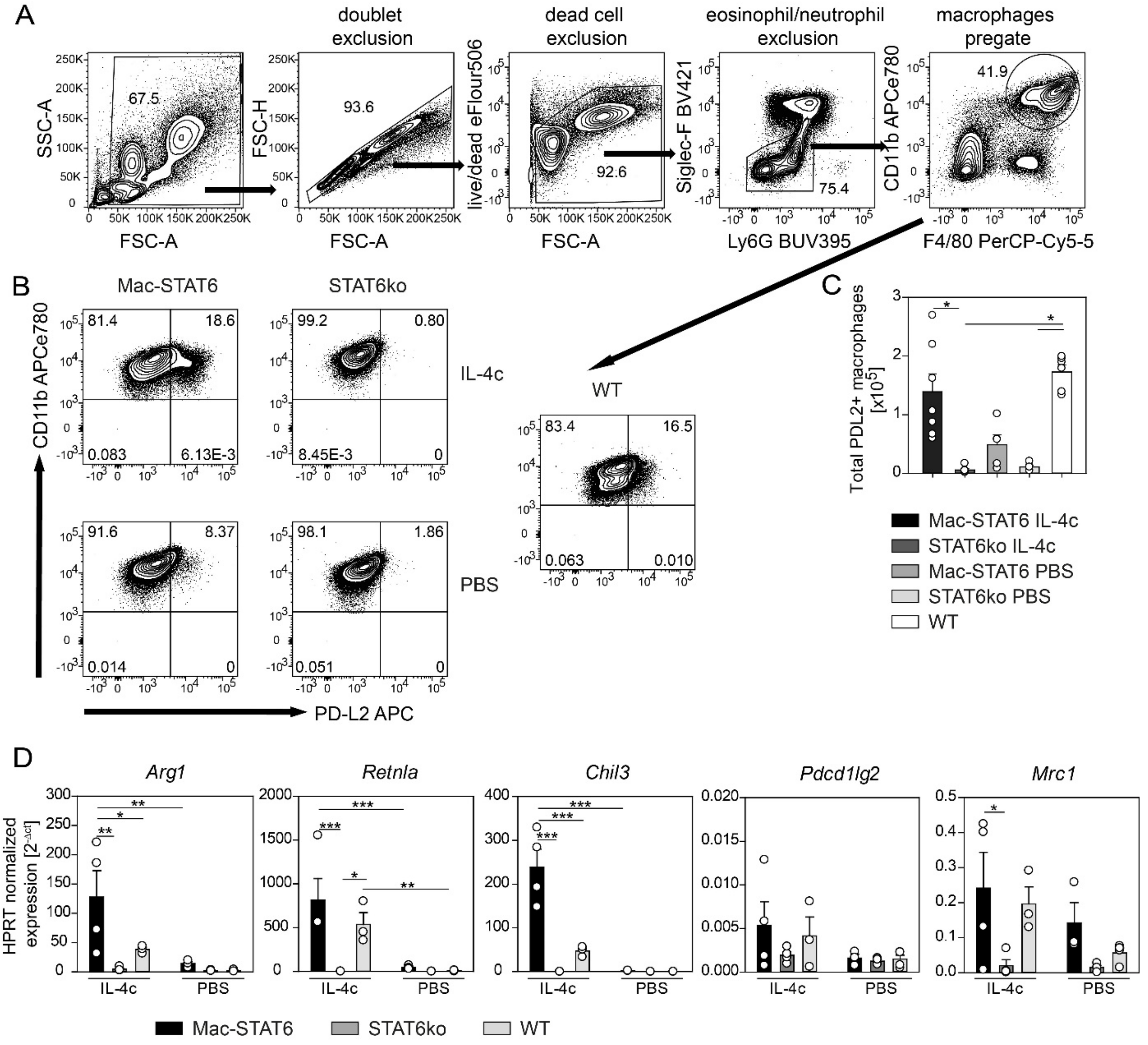

S2 Fig (related to Fig 1): AAMs in IL-4c-treated Mac-STAT6 mice. A) Gating strategy for flow cytometry analysis from peritoneal cavity (PEC) on day 9 after secondary *Hpb* infection. Doublets and dead cells were excluded as well as eosinophils and neutrophils. Cells were subsequently gated for CD11b and F4/80 double positive to define macrophages and these were then gated for PD-L2. B) Representative plots of $CD11b^+$ (APCeFlour780) versus $PD\text{-}L2^+$ (APC) cells in the PEC from Mac-STAT6 and STAT6ko or WT mice injected with IL-4c or PBS. C) Mean + SEM of total $PD\text{-}L2^+$ cells pooled from five to seven mice per group from three independent experiments. Statistical significance was determined by Kruskal-Wallis with Dunn's *post-hoc* testing. *$p < 0.05$. D) Mean + SEM of *HPRT* normalized expression of *Arg1*, *Retnla*, *Chil3*, *Pdcd1lg2* and *Mrc1* in sort-purified PEC macrophages. Naïve mice were treated with IL-4c *i.p.* on day 0 and 2 and sacrificed on day 4. PEC macrophages were sorted as $CD11b^+$ $F4/80^+$ cells and purity of sorted cells was >90%. Data are pooled from three to four mice per group from four independent experiments. Statistical significance was determined by Two-Way-ANOVA with Holm-Sidak *post-hoc* testing. ***$p < 0.001$; ** $p<0.01$; *$p < 0.05$.
