# Supplementary material for: Th2-dependent STAT6-regulated genes in intestinal epithelial cells mediate larval trapping during secondary Heligmosomoides polygyrus bakeri infection
Source: PLoS Pathog. 2023 Apr 5;19(4):e1011296. doi: 10.1371/journal.ppat.1011296 (PMC10109486; doi:10.1371/journal.ppat.1011296)
Supplement: S3 Fig — (PDF) [file ppat.1011296.s004.pdf]

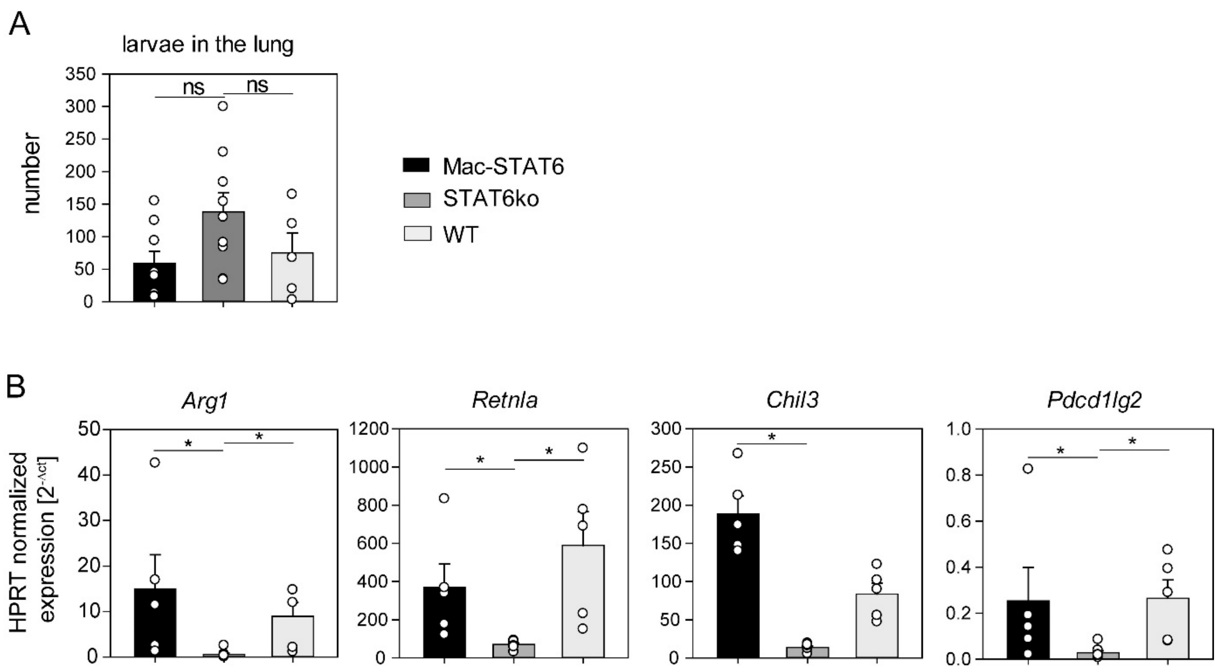

S3 Fig (related to Fig 2): *Nippostrongylus brasiliensis* (*Nb*) infected Mac-STAT6 mice. A) Mean + SEM of number of larvae migrated out of lungs from day 2 after secondary *Nb* infection. Data are pooled from two independent experiments and five to nine mice per genotype. B) Mean + SEM of *HPRT* normalized expression of *Arg1*, *Retnla*, *Chil3* and *Pdcd1lg2* in total lung tissue on day 2 after secondary *Nb* infection. Data are pooled from two independent experiments and display five to nine mice per genotype. A-B) Statistical significance was determined by One-Way ANOVA with Holm-Sidak *post-hoc* testing or, if normality or equal variance were not given, by Kruskal-Wallis with Dunn's *post-hoc* testing. ***p < 0.001; *p < 0.05.
