# Supplementary material for: Th2-dependent STAT6-regulated genes in intestinal epithelial cells mediate larval trapping during secondary Heligmosomoides polygyrus bakeri infection
Source: PLoS Pathog. 2023 Apr 5;19(4):e1011296. doi: 10.1371/journal.ppat.1011296 (PMC10109486; doi:10.1371/journal.ppat.1011296)
Supplement: S4 Fig — Antibody and germinal center response of Hpb infected Mac-STAT6 mice. (PDF) [file ppat.1011296.s005.pdf]

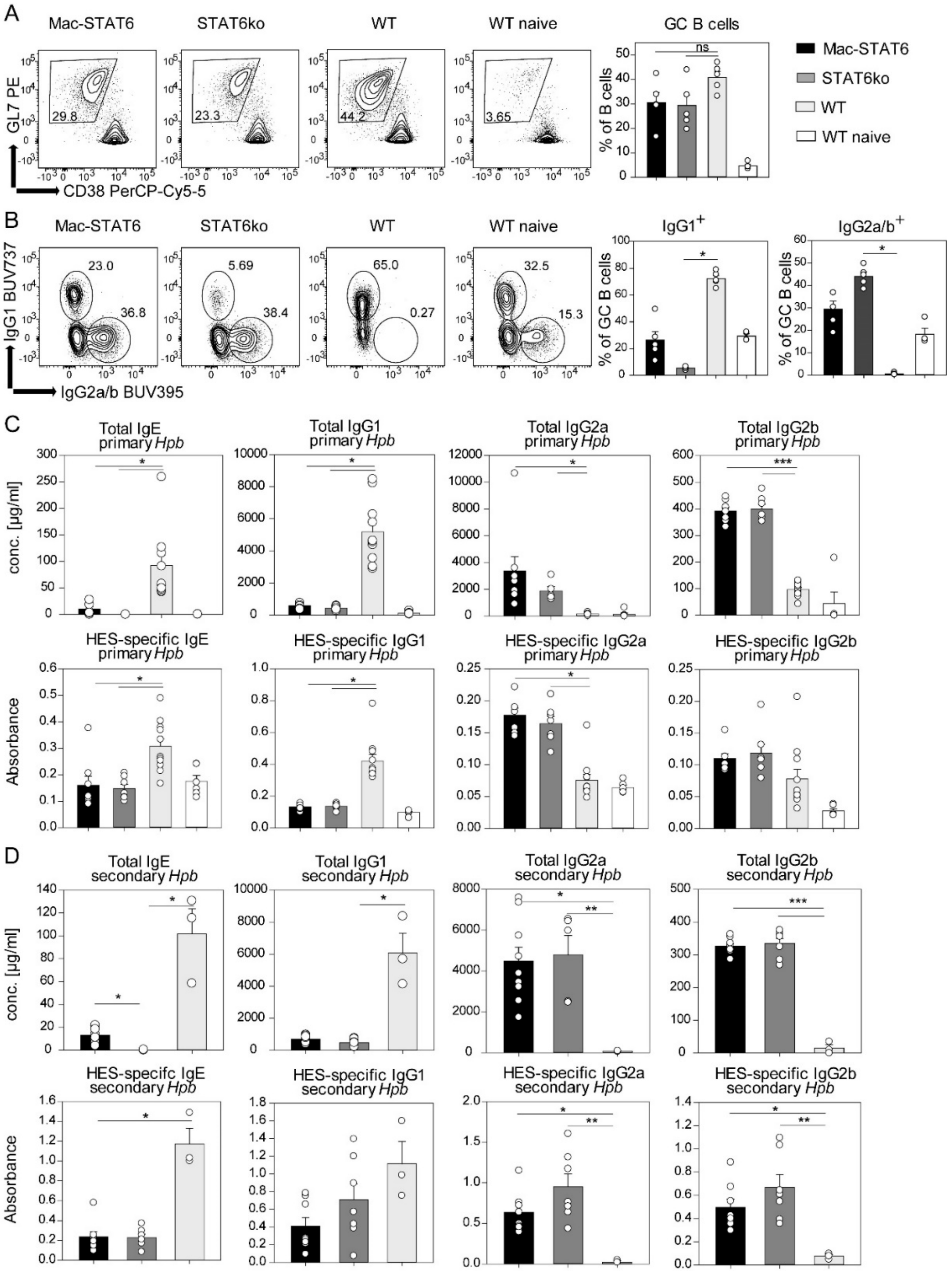

S4 Fig (related to Fig 2): Antibody and germinal center response of *Hpb* infected Mac-STAT6 mice. A) Representative plots and percentage of GC B cells (GL7$^+$CD38$^-$) in mLN of Mac-STAT6, STAT6ko and WT mice on day 14 after primary *Hpb* infection and naïve WT mice. Pre-gated on CD19$^+$B220$^+$ out of living cells. Mean + SEM of percentage of GC B cells of all B cells from four to five mice per group and two independent experiments. B) Representative plots and percentage of IgG1$^+$ and IgG2a/b$^+$ GC B cells. Pre-gated on GC B cells (GL7$^+$CD38$^-$) out of CD19$^+$B220$^+$ and living cells. Mean + SEM of percentage of IgG1$^+$ or IgG2a/b$^+$ of GC B cells from four to five mice per group and two independent experiments C) Total and Heligmosomoides excretory/secretory antigen (HES)-specific serum IgE, IgG1, IgG2a and IgG2b of Mac-STAT6, STAT6ko and WT mice on day 14 after primary *Hpb* infection and naïve WT mice. Mean + SEM antibody concentration (total) or absorbance (HES-specific) of six to eleven mice per genotype from three independent experiments is displayed. D) Total and HES-specific serum IgE, IgG1, IgG2a and IgG2b of Mac-STAT6, STAT6ko and WT mice on day 9 after secondary *Hpb* infection. Mean + SEM of measured concentration (total) or absorbance (HES-specific) of three to eight mice per genotype from three independent experiments is displayed. A-D) Statistical significance among infected groups was determined by One-Way ANOVA with Holm-Sidak *post-hoc* testing or, if normality or equal variance were not given, by Kruskal-Wallis with Dunn's *post-hoc* testing. ***$p < 0.001$; **$p < 0.01$; *$p < 0.05$.
