# Supplementary material for: Th2-dependent STAT6-regulated genes in intestinal epithelial cells mediate larval trapping during secondary Heligmosomoides polygyrus bakeri infection
Source: PLoS Pathog. 2023 Apr 5;19(4):e1011296. doi: 10.1371/journal.ppat.1011296 (PMC10109486; doi:10.1371/journal.ppat.1011296)
Supplement: S5 Fig — Gating strategy for flow cytometry analysis. (PDF) [file ppat.1011296.s006.pdf]

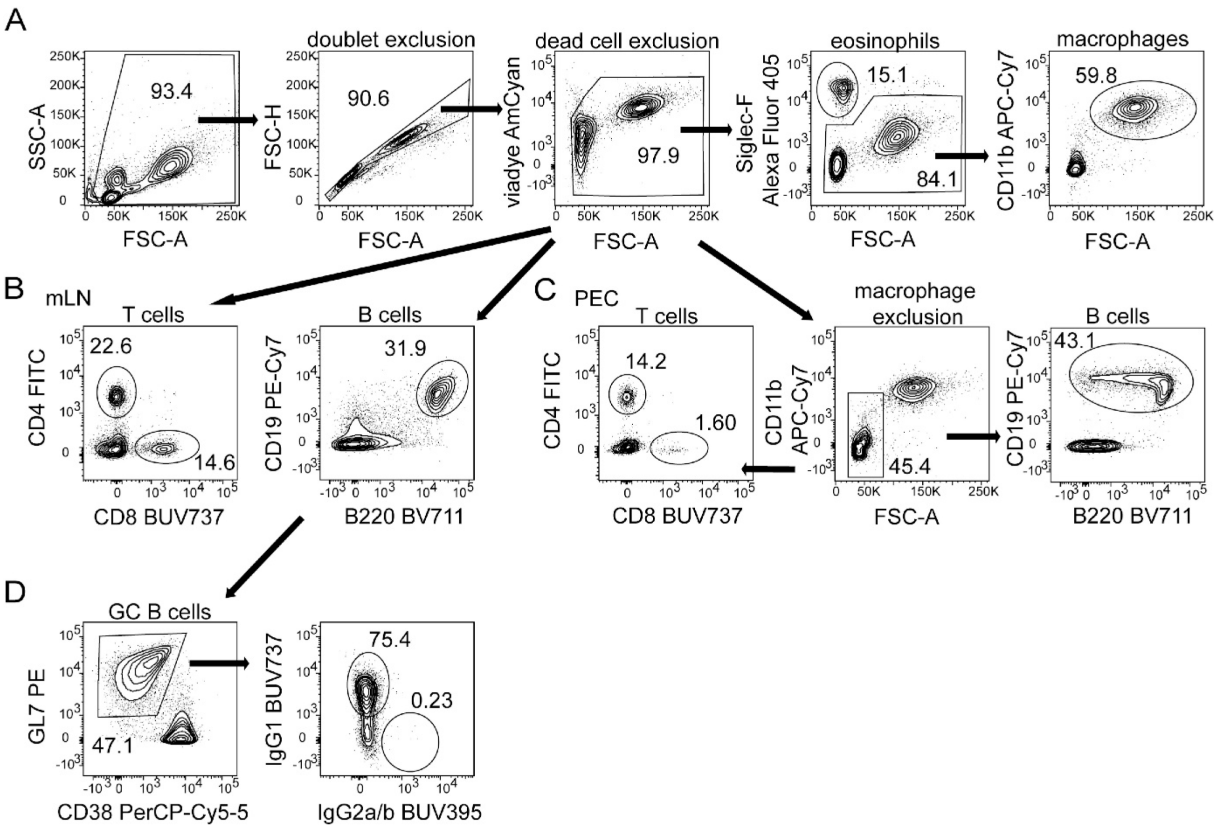

S5 Fig (related to Fig 2): Gating strategy for flow cytometry analysis. A) Representative gating strategy for analysis of PEC and mLN cells of *Hpb* infected mice. Doublets and dead cells were always excluded as first steps. Eosinophils in PEC and mLN were gated as Siglec-F$^+$ out of the live cell gate. Macrophages in PEC and mLN were gated as CD11b$^+$ cells after eosinophil exclusion. B) T cells (CD4$^+$ and CD8$^+$) and B cells (CD19$^+$B220$^+$) in the mLN were directly gated out of the live cell gate. C) For PEC T cells and B cells, additionally CD11b$^+$ macrophages were excluded after dead cell exclusion. D) For isotype stainings of germinal center (GC) B cells, it was gated on B cells (CD19$^+$B220$^+$) after dead cell exclusion, followed by GC B cells (GL7$^+$CD38$^-$) and the isotypes (IgG1 and IgG2a/b).
