# Supplementary material for: Th2-dependent STAT6-regulated genes in intestinal epithelial cells mediate larval trapping during secondary Heligmosomoides polygyrus bakeri infection
Source: PLoS Pathog. 2023 Apr 5;19(4):e1011296. doi: 10.1371/journal.ppat.1011296 (PMC10109486; doi:10.1371/journal.ppat.1011296)
Supplement: S6 Fig — Immune cell compartments in PEC and mLN of VillinCre_STAT6vt mice. (PDF) [file ppat.1011296.s007.pdf]

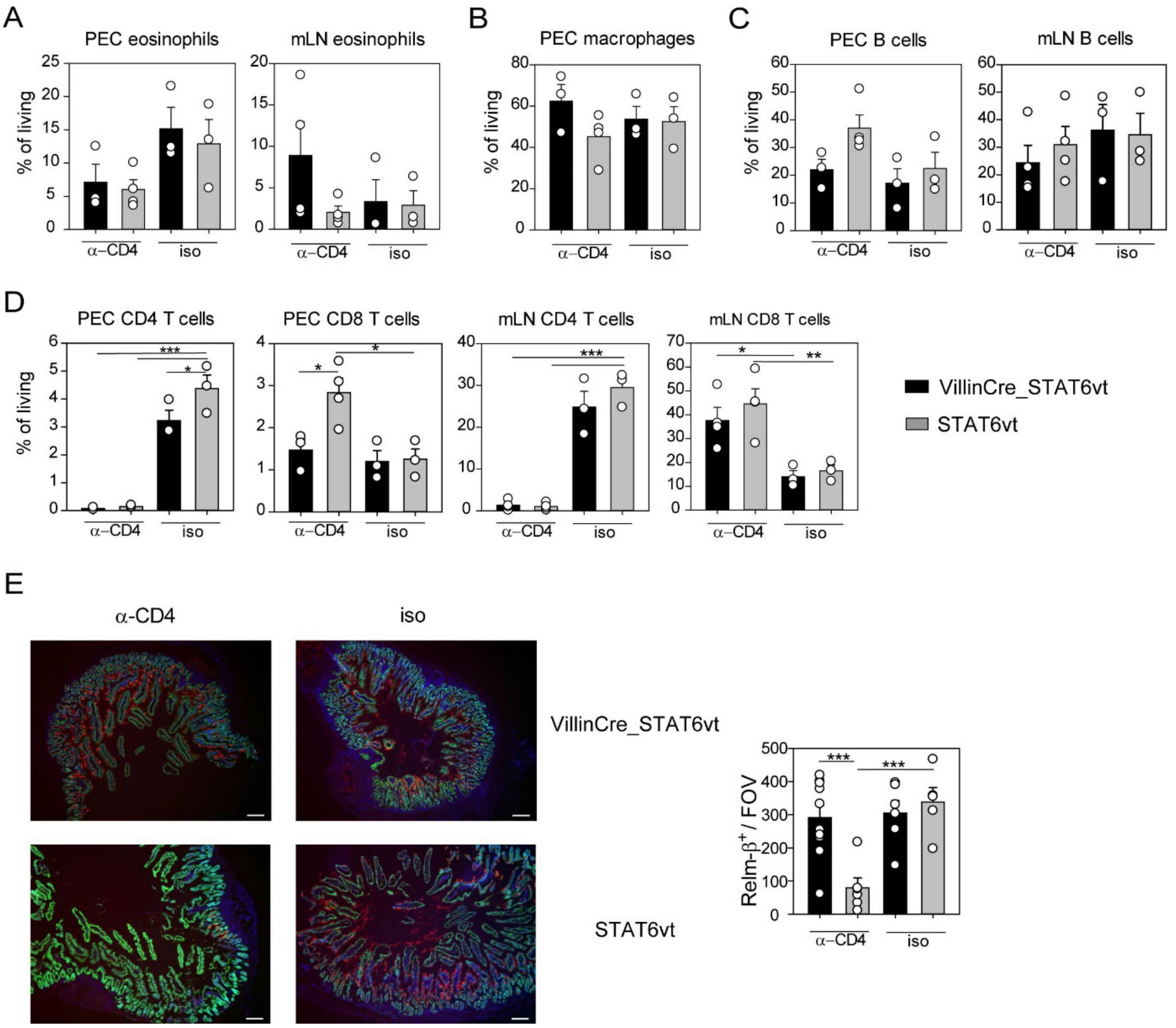

S6 Fig (related to Fig 4): Immune cell compartments in PEC and mLN of VillinCre_STAT6vt mice. VillinCre_STAT6vt and STAT6vt mice were secondarily infected with *Hpb* and $CD4^+$ T cells were depleted by *i.v.* antibody injection on day 0 and day 4 of secondary infection. Mice were sacrificed on day 9 after secondary infection and PEC and mLN cells were harvested for flow cytometry analysis. Mean + SEM of percentage of eosinophils (Siglec-F^+) (A), macrophages ($CD11b^+$) (B), B cells ($CD19^+B220^+$) (C) and $CD4^+$ and $CD8^+$ T cells (D) in PEC and mLN are displayed. Percentage of each population from all living cells was calculated and gating strategy as indicated in Fig S4. Data comprise three to four mice per group and are from one experiment. Statistical significance was determined by Two-Way ANOVA with Holm-Sidak *post-hoc* testing. ***p < 0.001; **p < 0.01; *p < 0.05. E) Representative pictures of Relm-β (red) with Epcam (green) and DAPI (blue) in histological sections from the small intestine day 9 after secondary *Hpb* infection. Scale bar is 200 µm. Quantified manually by counting Relm-β^+ IECs per field of view (FOV). Statistical significance was analyzed by Two-Way ANOVA with Holm-Sidak *post-hoc* testing. ***p < 0.001.
