# Supplementary material for: Th2-dependent STAT6-regulated genes in intestinal epithelial cells mediate larval trapping during secondary Heligmosomoides polygyrus bakeri infection
Source: PLoS Pathog. 2023 Apr 5;19(4):e1011296. doi: 10.1371/journal.ppat.1011296 (PMC10109486; doi:10.1371/journal.ppat.1011296)
Supplement: S7 Fig — Immune cell compartments in PEC and mLN of VillinCre_IL-4Rαfl/fl mice. (PDF) [file ppat.1011296.s008.pdf]

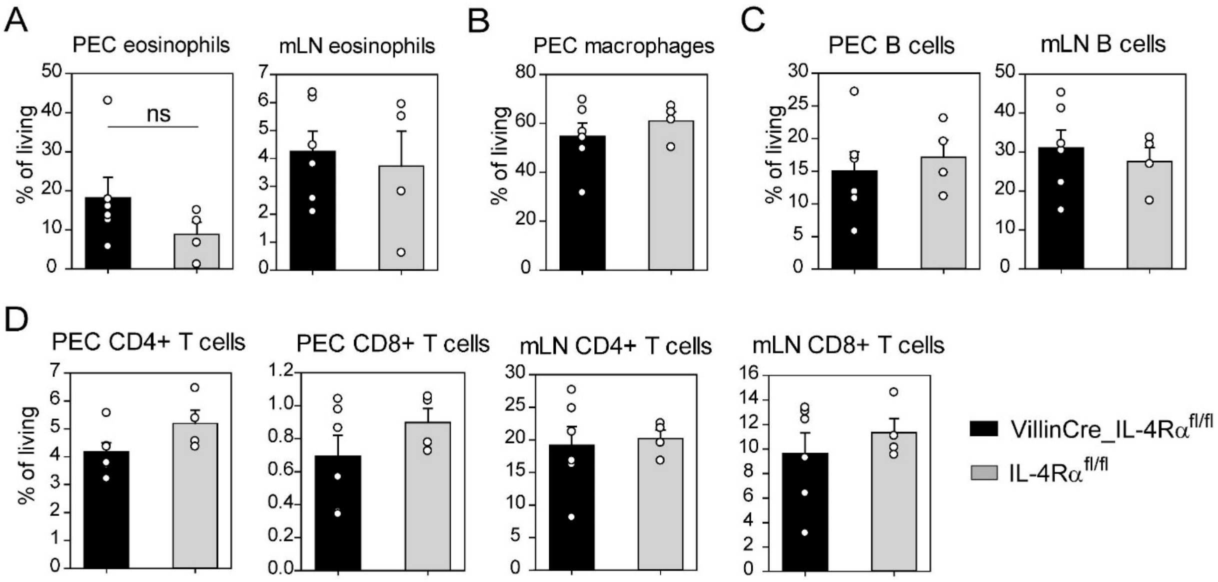

S7 Fig (related to Fig 5): Immune cell compartments in PEC and mLN of VillinCre_IL-4Rα$^{fl/fl}$ mice. VillinCre_IL-4Rα$^{fl/fl}$ mice and IL-4Rα$^{fl/fl}$ controls were sacrificed on day 9 after secondary *Hpb* infection and PEC and mLN cells were analyzed by flow cytometry. A) Mean + SEM of percentage of eosinophils (Siglec-F^{+}) (A), macrophages (CD11b^{+}) (B), B cells (CD19+B220+) (C) and CD4^{+} and CD8^{+} T cells (D). Percentages were calculated from total living cells and the gating strategy is indicated in Fig S4. A-D) Data in bars are pooled from four to six mice of two independent experiments. Statistical significance was analyzed by Student's t-Test, however no significant differences were found in any of the analyzed populations between VillinCre_IL-4Rα$^{fl/fl}$ mice and Cre^{-} littermate controls (IL-4Rα$^{fl/fl}$).
