# Supplementary material for: Th2-dependent STAT6-regulated genes in intestinal epithelial cells mediate larval trapping during secondary Heligmosomoides polygyrus bakeri infection
Source: PLoS Pathog. 2023 Apr 5;19(4):e1011296. doi: 10.1371/journal.ppat.1011296 (PMC10109486; doi:10.1371/journal.ppat.1011296)
Supplement: S8 Fig — Epithelial cell histology for 4-13Tko mice. (PDF) [file ppat.1011296.s009.pdf]

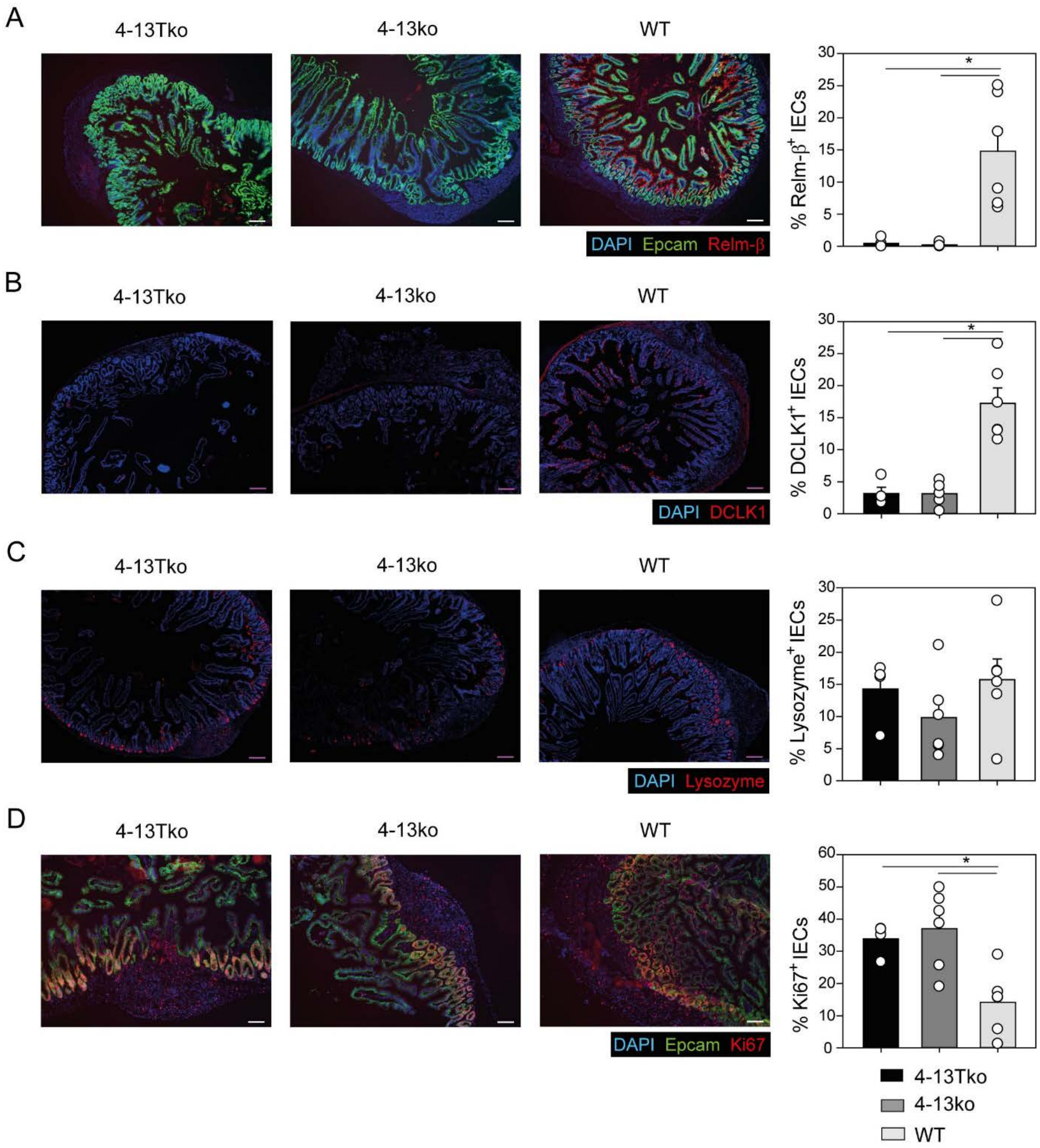

S8 Fig (related to Fig 6): Epithelial cell histology for 4-13Tko mice. Histological stainings and quantifications of small intestinal cross-sections day 9 after secondary *Hpb* infection for detection of Relm-β (red), Epcam (green) and DAPI (blue) (A) or DAPI (blue) and DCLK1 (red) (B) or DAPI (blue) and Lysozyme (red) (C) or Ki67 (red), Epcam (green) and DAPI (blue) (D). Quantification plots display Mean + SEM of percentage of Relm-β^+ or Ki67$^+$ of DAPI$^+$Epcam$^+$ epithelial cells (A, D) or DCLK1$^+$ or Lysozyme$^+$ of DAPI$^+$ cells (B, C). Representative picture and quantification for four to six mice per genotype and two independent experiments. Scale bar is 200 µm (A-C) or 100 µm (D). Statistical significance was determined by One-Way ANOVA with Holm-Sidak *post-hoc* testing or, if normality or equal variance were not given, by Kruskal-Wallis with Dunn's *post-hoc* testing. *$p < 0.05$.
