# Supplementary material for: Th2-dependent STAT6-regulated genes in intestinal epithelial cells mediate larval trapping during secondary Heligmosomoides polygyrus bakeri infection
Source: PLoS Pathog. 2023 Apr 5;19(4):e1011296. doi: 10.1371/journal.ppat.1011296 (PMC10109486; doi:10.1371/journal.ppat.1011296)
Supplement: Attachment — Submitted filename: Reviewer comments Westermann.docx [file ppat.1011296.s011.docx]

We thank the Editors and Reviewers for their helpful comments on our manuscript which we addressed as outlined in our point-to-point reply given below. We quantified the histology data, performed numerous additional experiments and edited the manuscript accordingly. The manuscript has now one additional main figure and four additional supplemental figures.

**Part I - Summary**
Please use this section to discuss strengths/weaknesses of study, novelty/significance, general execution and scholarship.

Reviewer #1: The article by Westermann et al describes the involvement of intestinal epithelial cells activation by IL-4/IL/13 in Heligmosmoides polygyrus trapping that occurs during secondary infection. In particular, they prove that macrophages arginase is not required for parasite trapping. This subject is of interest to the community, however I am not sure that the absence of macrophage requirement is proven nor does epithelial involvement in secondary protection is actually proven in the current version of the paper.

Reply: We did not conclude that macrophages in general are dispensable for larval trapping, but Arg1 expression is clearly not required and their differentiation to AAMs is not sufficient. We further add new data in the new Fig. 5 where we show that trapping is impaired in VillinCre_IL-4Ra-flox mice where only IEC are unresponsive to IL-4/IL-13 stimulation and AAMs are present in granuloma.

Number of repeats are sometimes too low for statistical analysis (example Fig3A) as 2 is not representative of a population.

Reply: We changed the presentation of the data in Fig3A to show two individual data points and the mean as a bar graph without error bars.

Many control are missing: For figure 1, WT+IL-4c should be presented as well for worm count and for histology. For figure 4, primary control would be useful, as their is already protection happening in those mice during primary infection (both for worm count and histology).

Reply: We believe that Fig 2 not Fig 1 is meant in the first part. Here, we would argue that WT mice are anyway trapping larvae so that additional administration of IL-4c would make no difference. The suggestion to include primary infections in Fig 4 is well-taken. However, since all other experiments were done during secondary infection when memory Th2 cells are present, we thought a better control would be to analyze VillinCre_IL-4Ra-flox mice during secondary infection. As expected, VillinCre_IL-4Ra-flox mice showed reduced larval trapping as compared to control mice after secondary infection while Arg1- and Relmα-expressing AAMs were still present in granuloma (new Fig. 5).

Reviewer #2: The manuscript explores further how infection with Hpb is controlled by recall immunity. Work from the Gause group has previously demonstrated an important role for AAM in immune memory against Hpb.
In this study the authors explore this relationship further. Using mice expressing only STAT6 in macrophages they identify that when type 2 sufficiency is only present in Macs recall immunity is impaired. Moreover in an Arg1 -/- setting recall immunity is maintained. This is contrary to previous studies using arignase inhibitors. However, this may be explained by maintenance of increased expression of other AAM associated markers.
The authors then address the role of epithelieal cells in contributing to the effector response that promotes recall immunity. The authors identify that an effective epithelial memory response can be launched where STAT-6 is inherently expressed in epithelial cells and CD4 T cells are depleted.
The authors then show that in the absence of IL-4 and IL-13 from T cells recall immunity is impaired.
Therefore recall immunity is driven by a epithelial response which requires CD4 T cell promotion.

Reviewer #3: The authors use a well-characterized model of helminth infection in mice, with H. polygyrus, to establish requirements for larval trapping in the submucosa following secondary infection, which has been reported to require alternatively-activated macrophages (AAMs) and Arginase-1 (Arg-1), signaling through IL4/13 and STAT6. Using transgenic mouse constructs they report that Arg1 is in their hands not required, and that STAT6 activation in AAMs not sufficient for trapping. Rather, STAT6 activation in intestinal epithelial cells is required.

The data are somewhat one-dimensional, for example evaluating helminth immunity solely by granulomas with and without trapped larvae (no adult worm recovery or egg counts), presenting immunohistological snapshots without quantification or image analysis. Overall it offers an incremental contribution rather than a conceptual advance.

Reply: The reason why we focused on an early time point after secondary infection and counted granulomas rather than adult worms in the lumen is based on the fact that we were specifically interested in the early protective effect caused by larval trapping rather than the well-studied later effect were goblet cells and smooth muscle cells cause the weep-and-sweep mechanism of adult worm expulsion. In the revised manuscript we include quantifications of the immunohistological stainings to provide a more objective view. It is well established that activated epithelial cells mediate expulsion of adult worms but their involvement in larval trapping in the submucosa is an unexpected finding. Further, it is largely believed that Arg1-expressing AAMs are critical for larval trapping. However, by using genetically Arg1-deficient mice we demonstrate that Arg1 is not required. We respectfully disagree with the notion that the manuscript shows only an incremental advance of our knowledge of protective immunity against helminths.

While this report shifts the focus towards an epithelial cell mechanism, there is no attempt to provide accompanying data on immune cell phenotypes in the different settings, and the paper does not advance far at any molecular level other than measuring the “usual suspects”. In particular, the authors miss the opportunity of using these mouse constructs (particularly the VillinCre-STAT6vt) for gene expression profiling and identification of the pathway within the epithelial population associated with immunity.

Reply: We apologize that we could not yet identify the molecular pathway(s) of epithelial cell-mediated larval trapping in the submucosa. However, we added now FACS data from PEC and mesenteric LN for some experiments to show the frequencies of distinct immune cells. Unfortunately, we had technical difficulties to isolate high quality RNA or viable cells from intestinal tissue of infected mice so that we could not do RNAseq or FACS analysis.

**Part II – Major Issues: Key Experiments Required for Acceptance**
Please use this section to detail the key new experiments or modifications of existing experiments that should be absolutely required to validate study conclusions.

Generally, there should be no more than 3 such required experiments or major modifications for a "Major Revision" recommendation. If more than 3 experiments are necessary to validate the study conclusions, then you are encouraged to recommend "Reject".

Reviewer #1: - Antibodies have previously been shown to be important for trapping of Heligmosmoides (Hp) during secondary infection (10.4049/jimmunol.1401645). The author supplement their Mac-STAT6 with IL-4c to activate macrophage into "M2" however, this is known to not be sufficient for trapping of Hp larvae. Have the author verified if the antibody response against Hp is normal in their Mac-STAT6 mice, as a defect in antibody response would certainly explain why the mice fail to trap the parasite regardless of the M2 polarisation. Of note, to our knowledge IL-4c supplementation in a naive mice has not been shown to be sufficient for Hp trapping (the protocol the authors refer to is for macrophage activation in the lungs). This control naive mice + IL-4c+ Hp is required both for worm count and for histology before being able to conclude that STAT6 activation of macrophage is not sufficient for parasite trapping.

Reply: The question whether the antibody response is affected in Mac-STAT6 mice is well taken. In the mentioned Esser-von-Bieren et al. publication the authors have shown that IgG2a/c rather than IgG1 antibodies are activating macrophages via FcgRI in vitro to express Arg1 and reduce Hp larval motility. We analyzed now the antibody isotypes in the serum of Hp-infected Mac-STAT6, STAT6ko and WT mice. Mac-STAT6 and STAT6ko mice had no general defect in Hp-specific antibody production but produced more IgG2a and IgG2b and less IgG1 and IgE compared to WT mice (new Suppl Fig 3). Therefore, we conclude that impaired trapping in Mac-STAT6 mice cannot be attributed to a defect in IgG2a or IgG2b production.

Urban and Finkelman have shown that naïve mice + IL-4c + Hp do not trap larvae in primary infection when IL-4c is given from d0 to d6 after infection. However, when given from d10 to d16 when adults are present in the intestinal lumen, IL-4c promotes worm expulsion during primary infection (Urban et al. (1995) J Immunol 154:4675). We cite this finding in our revised manuscript (ref. 34).

In FigS1, the authors actually report only 18.6% of peritoneal macrophage activated after IL-4c, which is really low. Only PDL-2 is used as activation marker (and not actually used anywhere else in the article), how are the classical M2 markers behaving :ARg1, RELMa, STAT6P ... As the IHC pictures are not presented per channel but only as merged, it is difficult to conclude if the macrophage are even activated in the STAT6MAc+IL4c.

Reply: We performed now RT-PCR analysis of sorted PEC macrophages with more AAM markers and show that Mac-STAT6 mice are not impaired in generating AAMs upon IL-4c treatment. In addition, we performed secondary infection with *N. brasiliensis* and found normal AAM marker expression in the lung of Mac-STAT6 and WT but not STAT6ko mice (new Suppl Fig 2). We also quantified RELMa-expressing macrophages in Fig. 2.

- Figure 3, a quantification of macrophage number in the mucosa, based on histology or flow cytometry to validate that ARg1 depletion is not affecting macrophage recruitment is necessary to reach conclusion. One could quantify peritoneal macrophages as a surrogate.

Reply: We add now quantification of macrophages for Fig 3C. The total number of macrophages in granuloma and the frequency of Relma+ macrophages was comparable in Tie2Cre_Arg1^fl/fl^ and Arg1^fl/fl^ mice.

- The use of the Villin_creSTAT6vt for secondary infection is very confusing. Indeed, the authors have previously shown that trapping takes place in those mice during primary infection and to a similar extent to what is reported here with the isotype control (about 60 granulomas with larvae). There seems to be no further protection due to reinfection. As such I fail to see the meaning of depleting CD4 T cells (previously shown to be required for secondary protection but not primary). Furthermore, the depletion of CD4Tcells in the control mice do not significantly impact the number of granulomas containing larvae.

Reply: One may hypothesize that activated IEC promote activation of Th2 cells which then secrete IL-4 to activate AAMs required for larval trapping in granulomas. Therefore, we depleted CD4 T cells to test this hypothesis. In the CD4 T cell-depleted controls we actually do see a 50% reduced number of trapped larvae (although not significant). This is consistent with previous findings showing that anti-CD4 treatment only before secondary infection results in impaired protection (Urban et al. (1991) Exp Parasitol 73:500, Anthony et al. (2206) Nat Med 12:955)

The conclusion of the authors "223 T cells are essential for larval trapping and activation of IECs in secondary Hpb infection." is not currently substantiated by their results.
The authors further state "However, the need for CD4+ 224 T cells could be overcome by
225 expression of a constitutively active form of STAT6 in IECs, emphasizing the prominent role
226 of STAT6-regulated genes in IECs in contributing to larval trapping", however they do not analyse what is the activation of those IECs, or their number. IHC of at least RELmb and an epithelial marker should be presented.
In absence of T cells, the author reports stronger RELmb expression, which contraditcs their claim that T cells are required for activation of IECs.

Reply: The last sentence is not correct as we show lower *Retnlb* expression in CD4 T cell-depleted control mice (grey bar in Fig 4D). To the first part: We include now a new data set from VillinCre_IL-4Ra-flox mice to show that lack of IL-4Ra signaling in IEC results in impaired larval trapping and blunted activation of IEC as shown by RELMb histology and RT-PCR analysis but unaltered AAMs in granuloma (new Fig 5).

- Finally, the authors show that IL-4/IL-13 derived from T cells is required for protection. While this is well clear, they also state that this is required for IEC activation. However, here they only report total gene expression of RELMb in the intestine, which is not satisfactory to fully describe an epithelial activation. Could the authors look at proliferation of epithelial cells surrounding the larvae in the granuloma, show that those cells upregulate RELMb maybe even Phospholipase A2, which has already been shown to be important for protection during reinfection. RELMb role in primary expulsion has been established but not in trapping of the larvae during secondary. The authors could prove that RELMbKO mice are not protected during reinfection.

Reply: We agree that the evidence for IEC activation in the old Fig 5 was rather poor. Therefore, we include now histology for RELMb (goblet cells), DCLK1 (tuft cells), Lysozyme (Paneth cells), Ki67 (cells in cell cycle) (Suppl Fig 7) and additional RT-PCR data for PLA2g1b which has been shown to contribute to protection during secondary Hp infection and Lysyl hydroxylase 2, a fibroblast-derived enzyme that promotes formation of collagen bundles. We actually don’t think that RELMb is involved in larval trapping as this molecule rather acts on adult worms in the luminal space of the small intestine.

Reviewer #2: The paper is really interesting but I feel could be hard for researchers to grasp.
The authors should include a graphical illustration which highlights the findings from this study in the context of input to recall immunity to Hpb.
This should also highlight the authors thoughts on the importance of AAM. This is the potentially contentious issue with this manuscript. The authors do explain and justify extremely well the reasons for this difference. However, use of an illustration to highlight how this study introduces new insights into the role of epithelial cells and macs in recall immunity really will promote the message of this study.

Reply: We thank the reviewer for this supportive comment. We actually had included a graphical abstract in the original submission. This should have been provided to all reviewers.

Reviewer #3: Major Issues

1. The authors do not present any further information on the cellular responses in the different mouse models, which would be important data to understand the mechanisms at play. For example are all other compartments (CD4, ILC2, eosinophils…) normal in the mice unable to trap larvae? What is different about the cellular response in the VillinCre-STAT6vt mice?

Reply: We tried several protocols to isolate viable cells for FACS analysis from intestines of infected mice – without satisfying results. However, we show now quantitative FACS data from PEC and mesenteric lymphnodes in Suppl Fig 5 and 6.

2. The authors entitle Figure 2 “AAMs are not sufficient for larval trapping”; however the authors do not show data to document that in vivo AAM development is equal in the WT and Mac-STAT6 mice, they rely on in vitro phenotypes as shown in Figure 1.

Reply: In addition to the in vitro data shown in Fig 1 we had quantified PD-L2-expressing PEC macrophages of IL-4c treated Mac-STAT6 and control mice (Suppl Fig 1C). To provide further evidence that AAM develop in Mac-STAT6 mice we (1) performed secondary *N. brasiliensis* infection and analyzed lungs for expression of AAM markers (Suppl Fig 2B) and (2) sorted PEC macrophages from IL-4c-treated Mac-STAT6 and control mice and performed RT-PCR analysis of AAM marker genes (Suppl Fig 1D).

3. Interpretation of the immunohistology sections relies on subjective judgement, which is questionable. For example, (lines 144-146) “Relm-a was abundantly expressed in IL4c-treated Mac-STAT6 mice” whereas comparison with the WT panel shows a very substantial reduction; hence it would appear (in contrast to the statement on lines 150-151) that both Relm-a and Arg-1 and Relm-a are compromised in the Mac-STAT6 mice. For objectivity, and also to ensure that the images are representative, quantification of large number of sections should be performed.

Reply: We performed now quantitative image analysis for Fig 2C/D, Fig 3C/D, Fig 4E, Fig 5B, Fig 6C, Suppl Fig 5E, and Suppl Fig 7. This was done by using the Fiji image software in most cases except for quantification of Relm-b+ cells (goblet cells) in Fig 5B and Suppl Fig 5E where we manually counted cells per field of view because the software had problems here.

4. In Figure 3 the authors use a genetic KO of arginase-1 to follow up on published work with the pharmacological inhibitor BEC (Anthony et al, 2006 Ref 7). They find however that these mice retain the ability to trap larvae, in contrast to the earlier study. It is unfortunate that the authors did not also include WT mice treated with BEC to establish if the different result is due to the model, or other extraneous factors such as the animal facility being used. (Again Figure 3C is not accompanied by any quantitative analysis).

Reply: We agree that it would have been nice to include BEC treatment in our study. However, commercially available BEC to be used at the 0,2% concentration in drinking water reported in the Anthony et al paper appears to be unaffordable. Fig 3C is now quantified.

5. Generally, IL4/13 and STAT6 signaling alter the development of intestinal stem cells towards different specialized cell types. Hence an important dimension missing from Figure 4 is any analysis of goblet, Paneth and tuft cells in the different experimental groups.

Reply: We have shown before that goblet, Paneth and tuft cells are increased in the SI of VillinCre_STAT6vt as compared to control mice (Schubart et al. Muc Immunol 12:413). But we include now further histological analysis in Suppl Fig 7 for these three epithelial cell types.

6. For Figure 5, the authors use IL4/13 KO mice, both as global and T cell-specific Kos to measure the impact on helminth immunity; as would be predicted, immunity is ablated in both. The insight offered by this new experiment is very limited, as in the absence of T cell cytokines from the outset of infection it is only to be expected that immunity will fail to develop ; it would be more useful to ascertain whether T cell cytokines are required at the time of challenge infection.

Reply: Although one may predict a critical role of T cell-derived IL-4/IL-13 for larval trapping during secondary Hp infection, this has to our knowledge not been shown before. Depletion of CD4 T cells (Urban et al. Exp Parasitol 73:500, Anthony et al. Nat Med 12:955) or IL-4 (Urban et al. PNAS 88:5513) during secondary Hp infection has been done before and both parameters were found to be important. But this did not allow to conclude that T cells are the critical source of IL-4. We further include now data from VillinCre_IL-4Ra-flox mice which showed impaired larval trapping during secondary Hp infection (new Fig 6). We again emphasize that we focused on trapping within the first week after infection rather than later protective effects mediated by the weep-and-sweep mechanism that acts on adult luminal worms.

**Part III – Minor Issues: Editorial and Data Presentation Modifications**
Please use this section for editorial suggestions as well as relatively minor modifications of existing data that would enhance clarity.

Reviewer #1: - line 104 : the authors state : "Contrary to expectations and for reasons that remain unclear, STAT6 in the BMDMs of Mac-STAT6 mice was not constitutively phosphorylated". It is unclear to me while constitutive activation of STAT6P would have been expected, as this is usually regarded as M2 polarisation marker.

Reply: The Mac-STAT6 mice express a modified version of STAT6 (named STAT6VT) in macrophages which has two amino acid changes. STAT6VT is constitutively phosphorylated by an unknown kinase when expressed in other cell types like intestinal epithelial cells or B cells (Haase et al. Eur J Immunol 50:558, Schubart et al. Muc Immunol 12:413). Therefore, we expected to observe spontaneous M2-polarization in Mac-STAT6 mice which was not the case. We revised the text to clarify this point.

- For all histology, could the author separate the channels, as it would help see colocalization.

Reply: Showing separate channels for all histology would be a lot of panels. We rather used image analysis software to quantify co-expressing cells.

- In figure 2C both in WT and in STAT6Mac there is a strong RELMa staining that do not colocolize with CD68, potentially of epithelial origin. However the authors then use RELMa as a marker of macrophage only as opposed to RELmb as a marker of IEC. Staining for EpCAM or related IEC marker might be helpful in the current setting.

Reply: This point is well taken. We performed now quantitative image analysis for CD68 and RELMa co-expressing cells instead of performing RELMa+EpCAM stainings (revised Fig. 2).

- Fig2D:Why is there Arg1 expression in STAT6KO mice ? Does this actually colocolize with a macrophage marker ? In IL4RKO mice, arginase stain has been previously reported to not be present (10.1371/journal.ppat.1003771). There also seems that there is less Arg1 around the larvae in the MACSTAT6+ IL4c that in the STAT6KO which is confusing. A quantification of the staining would be helping with interpretation. Outlining the larvae as well (as they are quite autofluorescent) would help as well.

Reply: Arg1 can also be induced by LPS or other stimuli and is not strictly dependent on STAT6 (e.g. El Kasmi et al. Nat Immunol (2008) 9:1399-1406). We quantified the histology data in Fig. 2D for cells co-expressing DAPI, CD68 and Arg1 or Relmα. The larvae are present in WT mice but not in Mac-STAT6 mice as the latter do not trap the larvae.

-There have been some recent papers with improve cell isolation protocol during Hp infection (Ferrer-Fon et al, 2020 elife; Bouchery et al, 2017, current protocol). Flowing the intestinal macrophage might thus give a better idea of their actual polarisation.

Reply: We tried these protocols but unfortunately still got very poor viability from tissue of secondarily Hp infected mice.

- Besides, AAMs, defined here by Relm-α and
180 CD68 co-expression, are surrounding the granuloma in a similar amount in mice of both
181 genotypes (Fig 3C, left panel.
Please quantify based on images.

Reply: We quantified the data now.

Reviewer #2: (No Response)

Reviewer #3: Minor Issues
1. Line 63 Reference 8 describes work with N brasiliensis not H polygyrus as stated here.

Reply: We added N brasiliensis to this sentence.

2. Lines 65-67 (and throughout the manuscript) – there is no mention of tuft cells

Reply: We added tuft cells and quantified tuft cells in Suppl Fig 7.

3. Lines 69-78 the text describing previous work lacks insight or detail and any key differences between the studies are not defined. At least 2 references are missing:
G. Chen, S. H. Wang, J. C. Jang, J. I. Odegaard and M. G. Nair: Comparison of RELMα and RELMβ Single- and Double-Gene-Deficient Mice Reveals that RELMα Expression Dictates Inflammation and Worm Expulsion in Hookworm Infection. Infect Immun, 84(4), 1100-1111 (2016) doi:10.1128/IAI.01479-15
H. M. Batugedara, J. Li, G. Chen, D. Lu, J. J. Patel, J. C. Jang, K. C. Radecki, A. C. Burr, D. D. Lo, A. R. Dillman and M. G. Nair: Hematopoietic cell-derived RELMα regulates hookworm immunity through effects on macrophages. J Leukoc Biol (2018) doi:10.1002/JLB.4A0917-369RR

Reply: We added these references as Ref. 20 and Ref. 21.

4. Line 79 diverse not divers

Reply: We corrected this typo.

5. Figure 1 B : why do most graphs show large effects but no significance?

Reply: We re-analyzed the data by ANOVA after log-transformation resulting in the expected significant differences.

6. Line 335 where not were

Reply: We corrected this typo.

7. Lines 395-403 Methods detailing B cell purification do not appear to be necessary as no data are shown.

Reply: We show Western Blot data from purified B cells in Fig 1A.
